# Enzymatically inactive caspase-1 mediates a proinflammatory phenotype in mice

**DOI:** 10.1186/1546-0096-13-S1-O53

**Published:** 2015-09-28

**Authors:** S Reinke, A Gocht, H Luksch, A Rösen-Wolff, S Winkler

**Affiliations:** 1 University Hospital Carl Gustav Carus, Technische Universität Dresden, Germany, Department of Pediatrics, Dresden, Germany

## Introduction

The proinflammatory enzyme caspase-1 belongs to the family of cystein proteases and plays a pivotal role in innate immunity. Classically, caspase-1 mediated proinflammatory signaling is thought to be dependent on its enzymatic activity. Interestingly, a number of patients carrying variants of the *CASP1* gene show proinflammatory symptoms like febrile episodes despite of reduced enzymatic activity of the caspase-1 protein resulting in decreased IL-1β production. *In vitro* investigations of this contradictory phenomenon revealed receptor interacting protein kinase 2 (RIP2) as a potential mediator between decreased caspase-1 activity and increased inflammation. In order to investigate the role of enzymatically inactive caspase-1 in an *in vivo* situation, we generated a mouse model expressing enzymatically inactive caspase-1.

## Objectives

The main objective of the study is to uncover the effects and mechanisms of enzymatically inactive caspase-1 in inflammatory signaling *in vivo.*

## Materials and methods

We generated two different mouse models of enzymatically inactive caspase-1 C284A using a BAC transgene and a conditional gene targeting approach. Successful integration was verified by PCR for the BAC transgene or by PCR and Southern Blot analyses for the gene targeting approach. Transgene expression was proven by qRT-PCR and Western Blot analyses of different tissues. In order to specifically investigate the effect of inactive caspase-1, the transgenic mice were crossed back on a caspase-1 knockout background. The influence of enzymatically inactive caspase-1 on inflammatory signaling was analyzed using a model of LPS-induced endotoxin-shock. Readout was change of body temperature and cytokine levels in the serum.

## Results

Following LPS-induced endotoxin shock, mice expressing enzymatically inactive caspase-1 (Casp1^-/-^/R26Casp1C284A^+/+^) showed a pronounced decrease of body temperature and increased levels of the proinflammatory cytokines TNF-α and IL-6 compared to caspase-1 knockout mice (Casp1^-/-^). However, the strongest decrease of body temperature was found in wildtype controls (Caspase-1^+/+^).

## Conclusion

Taken together, we show for the first time that enzymatically inactive caspase-1 is able to initiate proinflammatory signaling *in vivo*. This data is in line with several publications assuming a scaffold-function of caspase-1 independent of its enzymatic activity. However, the proinflammatory signaling mediated by enzymatically active caspase-1 seems to be stronger in the context of LPS-induced endotoxin shock. In order to uncover potential mediators and mechanisms of proinflammatory signaling initiated by enzymatically inactive caspase-1, gene expression profiling and secretome analysis are needed in the future.

This study was supported by the German Research Foundation (DFG, KFO 249).

